# Barriers and facilitators to early rehabilitation in mechanically ventilated patients—a theory-driven interview study

**DOI:** 10.1186/s40560-018-0273-0

**Published:** 2018-01-23

**Authors:** Shannon L. Goddard, Fabiana Lorencatto, Ellen Koo, Louise Rose, Eddy Fan, Michelle E. Kho, Dale M. Needham, Gordon D. Rubenfeld, Jill J. Francis, Brian H. Cuthbertson

**Affiliations:** 1 Department of Critical Care Medicine, Sunnybrook Health Sciences Centre, 2075 Bayview Ave, Toronto, Ontario M4N 3M5 Canada; 2 Institute of Health Policy, Management and Evaluation, University of Toronto, Toronto, Canada; 3 School of Health Sciences, City University London, London, UK; 4 Lawrence S. Bloomberg Faculty of Nursing, University of Toronto, Toronto, Canada; 5 Provincial Centre for Weaning Excellence, Toronto East General Hospital, Toronto, Canada; 6 Interdepartmental Division of Critical Care Medicine, University of Toronto, Toronto, Canada; 7 School of Rehabilitation Science, McMaster University, Hamilton, Ontario Canada; 8 Division of Pulmonary and Critical Care Medicine and Department of Physical Medicine and Rehabilitation, Johns Hopkins University, Baltimore, USA

**Keywords:** Rehabilitation, Critical illness, Intensive care, Quality improvement, Qualitative research, Health personnel

## Abstract

**Background:**

Despite a supportive evidence base and a push to implement, the uptake of early rehabilitation in critical care has been inconsistent. The objective of this study was to explore barriers and facilitators to early rehabilitation for critically ill patients receiving invasive mechanical ventilation.

**Methods:**

Using the Theoretical Domains Framework (TDF) of behavior change, we conducted semi-structured interviews exploring barriers and facilitators to early rehabilitation among four purposively sampled ICU clinician groups (nurses, rehabilitation professionals, respiratory therapists, and physicians). The TDF is a comprehensive framework of 14 “construct domains,” synthesized from 33 theories of behavior that was developed to study determinants of behavior and to design interventions to improve evidence-based healthcare practice. A topic guide was developed and piloted based on the TDF and expert knowledge. Interviews were audio-recorded and transcribed verbatim. Transcripts were content analyzed by coding items into domains and then synthesized into more specific, over-arching themes or “beliefs.” An expert consensus group used structured decision rules to classify beliefs as high, moderate, or low in importance.

**Results:**

We interviewed 40 stakeholders from the four clinician groups and identified 135 separate beliefs. Of these, 19 were classified as high, 40 as moderate, and 76 of low importance as barriers or facilitators. All beliefs classified as highly important fell within one of seven TDF domains: skills, social/professional role and identity, beliefs about capabilities, beliefs about consequences, environmental context/resources, social influences, and behavioral regulation. Beliefs of lower importance fell under the following seven domains: knowledge; optimism; reinforcement; intention; goals; memory, attention, and decision processes; and emotion. Quantitative differences in stated beliefs about early rehabilitation between professional groups were not common.

**Conclusions:**

This study identified important barriers and facilitators to early rehabilitation in critical care patients. Domains identified as important should be considered when designing interventions to increase uptake of early rehabilitation.

**Electronic supplementary material:**

The online version of this article (10.1186/s40560-018-0273-0) contains supplementary material, which is available to authorized users.

## Background

Traditionally, critical illness involved a period of deep sedation and immobility. However, deep sedation can be harmful [1, 2], and critical illness is associated with significant muscle atrophy and weakness [3, 4]. Physical rehabilitation, initiated early in the course of critical illness, is an active area of research within critical care. Observational studies to date have demonstrated the safety and feasibility of early rehabilitation with critically ill patients [5–8] and successful implementation in single centers [9, 10]. Randomized trials, summarized in a recent systematic review [11], as well as two randomized trials, demonstrate improved patient-centered outcomes with early rehabilitation strategies [11–13].

Despite a supportive evidence base and a significant push to implement such practices [14–16], uptake of early rehabilitation has been at best inconsistent. Point prevalence studies have documented low levels of involvement of physical therapists in the intensive care unit (ICU) and low rates of implementation of rehabilitation [17, 18]. Prior work studying barriers to implementation of early rehabilitation strategies in the ICU has focused on resource issues and concerns about patient tolerance and safety primarily from the perspective of physical therapists and physicians, with minimal input from nurses and no input from respiratory therapists.

There is broader evidence that the translation of complex, evidence-based interventions into clinical practice is often a slow and haphazard process [19, 20]. It has been argued that implementation and clinician behavior change may be facilitated through the application of theory to systematically identify the hypothesized causal mechanisms and factors influencing clinical practice [21, 22]. The Theoretical Domains Framework (TDF) [23, 24] of behavior change synthesizes constructs from 33 behavior change theories into 14 “construct domains,” or clusters of related constructs that may explain practice change or the absence of change (see Additional file 1). It has been applied as a framework for developing questionnaires and interview topic guides across a range of clinical contexts to systematically explore the barriers and facilitators to clinician behavior change [25, 26]. Each domain represents a range of related constructs that may influence clinician behavior. For example, the domain “social influences” encompasses overlapping constructs such as professional identity, boundaries, confidence, leadership, and organizational culture/climate.

This study explored clinician-reported barriers and facilitators to early physical rehabilitation in critically ill patients receiving invasive mechanical ventilation. Additionally, the study assessed relative importance of the identified barriers to early rehabilitation.

## Methods

### Study design

This was a semi-structured interview study, based on the TDF, of ICU clinicians’ perceptions of barriers and facilitators to early rehabilitation.

### Participants

Participants were purposively sampled from one of four clinician groups: critical care nurses, physicians, respiratory therapists, and rehabilitation professionals (physical therapists and occupational therapists) to achieve diversity in terms of years of experience, academic versus non-academic work environment, leadership position, ICU size, and country of practice (USA/Canada). Participants had to work as independent practitioners primarily caring for adult patients and were required to identify critical care as a focus in their practice.

To achieve the goals of maximum variability sampling described above [27], we recruited from the “ICU Recovery Network” (IRN), an online interest group of clinicians interested in critical care rehabilitation and recovery from critical illness, and multiple professional associations and collaborative research groups.

### Development of topic guide

A semi-structured interview topic guide was developed based on the TDF and expert knowledge from the author group. At least one question for each of the 14 domains of the TDF was included. The interview guide was drafted by two critical care clinicians (SLG and BHC) and two health psychologists with expertise in the TDF (JF and FL). Following feedback from the wider investigator team and piloting with one clinician from each of the four clinical groups, questions were revised to minimize duplication and enhance clarity, clinical relevance, and completeness.

To assess the extent to which the questions were likely to elicit responses related to each domain, the questions were independently coded into domains by a health psychologist with expertise in the TDF (AP). The reliability of this coding was assessed with Cohen’s kappa [28]. The final interview topic guide is available in Additional file 2.

All interviews were conducted by a single member of the study team (EK) by telephone. All interviews were audio-recorded, transcribed verbatim, checked for accuracy, and anonymized. EK had prior experience with semi-structured interviewing and received additional context-specific training through detailed review of and feedback on pilot interviews provided by ICU clinicians (SG and BC) and health psychologists with experience in semi-structured interviewing (FL and JF).

### Analysis

Using NVivo (version 10), data were analyzed using content analysis [29]. All participant utterances within each transcript were assigned to TDF domains by one investigator (SG). Responses could be allocated to more than one domain. Initially, a sub-sample of 10% of transcripts was independently coded by a second investigator (FL) to assess inter-rater reliability using Cohen’s kappa. If Cohen’s kappa was less than 0.7, coding strategies were reviewed with a plan to review a further 10% of transcripts as necessary. Discrepancies were resolved through consultation with additional team members (BC, JF).

Following initial coding, participants’ responses across transcripts were compared within each domain. Responses that were thematically similar were grouped to inductively identify a “belief” relevant to early rehabilitation. Additional detail to illustrate these methods is provided in Additional file 3. Analysis of interviews was continued until saturation was achieved, with at least two additional interviews per group analyzed beyond that point [30]. We planned to analyze approximately equal numbers of participants in each group to simplify quantitative comparisons.

An expert consensus group comprising the wider investigator team met to review all domain and belief coding for clinical and theoretical face validity. In addition, to establish importance, the group collectively reviewed each belief with the following considered as evidence of importance: (1) high frequency of belief (more than half the participants), (2) any participant expression of importance (e.g., “it’s critical to educate the staff”), (3) discord among participants about belief as a barrier or facilitator, (4) differences between clinician groups in frequency by at least five participants, and (5) whether a belief was expressed spontaneously versus prompted by a direct question. Theoretical and empirical work supports the use of multiple methods to establish importance in barriers work [31]. This approach to understanding importance has been used in prior TDF work [32]. Beliefs were classified as “low importance” if zero or one of the five criteria was met and of moderate importance if two criteria were met. All other beliefs were classified as high importance. A detailed explanation of the criteria for assessing importance is found in Additional file 4.

### Ethical considerations

This study was reviewed and approved by the Research Ethics Board at Sunnybrook Health Sciences Centre (Reference # 015-2014). Participation in the study was voluntary, and all data were anonymized. Telephone consent was obtained from all participants and recorded by the research assistant (EK) on written consent forms for each participant.

## Results

### Participants

Forty participants were included. The denominator of potential participants is unknown because of the use of public listservs and email lists without known numbers. Saturation was achieved for each of the four clinician groups by a maximum of eight interviews. Interviews lasted a mean of 46 min (range 20–80 min). Participant details are shown in Table 1.

**Table 1:** Participant characteristics

		Frequency (*N* = 40)
Professional group	Rehabilitation professional	10
	Nurse	10
	Respiratory therapist	10
	Physician	10
Professional leadership role	Yes	18
	No	22
Country of employment	Canada	23
	USA	17
Type of institution	Academic health sciences center	25
	Community teaching hospital	10
	Community non-teaching hospital	5
Number of ICU beds	< 10	3
	10–20	10
	21–50	14
	> 50	13
Years since graduation	≤ 5	8
	6–10	10
	> 10	22
Years of ICU experience	≤ 5	10
	6–10	11
	> 10	19

### Inter-rater reliability

Cohen’s kappa for blinded assignment of questions to TDF domains was 0.89. Cohen’s kappa for duplicate coding of transcripts into TDF domains was 0.74.

### Results by domain

A total of 135 beliefs related to early rehabilitation were identified across the 14 domains of the TDF. Of these, 19 were classified as high importance, 40 of moderate importance, and 76 of low importance as barriers or facilitators of early rehabilitation.

**Fig. 1:**
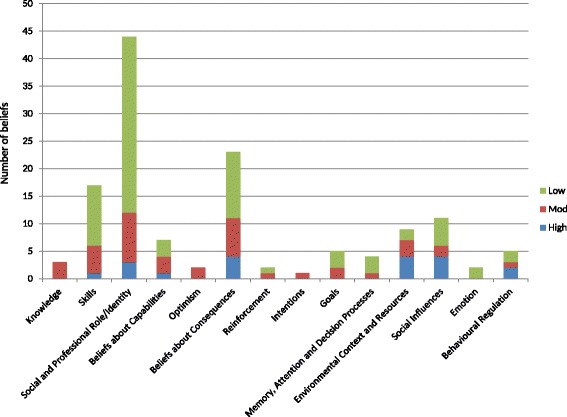
Number of unique beliefs identified in each domain and assigned importance

All beliefs classified as highly important fell within one of seven domains from the TDF: skills, social/professional role and identity, beliefs about capabilities, beliefs about consequences, environmental context and resources, social influences, and behavioral regulation. As shown in Fig. 1, domains with high importance beliefs also contained the highest number of beliefs identified. Beliefs of high importance are shown in Table 2 with exemplar quotations. Beliefs of moderate importance are shown in Additional file 5. High importance beliefs are elaborated below.

**Table 2:** Important beliefs according to the domain of the TDF

Belief (number of participants endorsing belief)	Excerpt from the interview
Skills domain	
Skills for early rehabilitation are developed by working with experienced colleagues. (12)	“I’d say what’s helped me a lot is mentorship, working with a more skilled ICU therapist that’s been doing things for a little bit longer...” (PT)
Social and professional role/identity domain	
Leadership has an important role in early rehabilitation. (22)	“… I work so closely with the nurses that if the nurses weren’t on board it would be very difficult to do early mobility.” (PT)
Physicians have an important role in identifying appropriate patients for early rehabilitation. (16)	“…we all try to be there and try to suggest or support rehabilitation, but in the end, again it ends up being a physician call when the physios get involved.” (RT)
Physicians have an overall leadership role in early rehabilitation. (7)	“And I like to talk to the doctors and have it spelled out to me what’s okay, what’s not okay with that patient, if it’s not clear from looking at the chart.” (PT)
Beliefs about capabilities domain	
Early rehabilitation is challenging. (22)	“I would describe it as very important but also very challenging, to get the people up at such a … critical time as their care.” (PT)
Beliefs about consequences domain	
Early rehabilitation decreases muscle atrophy or reduces weakness. (31)	“It limits or even reverses weakness and muscle wasting.” (PT)
Early rehabilitation affects long-term physical function. (22)	“I would say the most important goal… is to improve patient outcomes …also functional outcomes in the long term.” (MD)
Early rehabilitation affects the mental health of the patient. (27)	“…it gives them a huge sense of psychological and psychiatric benefits, because I think laying in that bed, day after day, it can put a tremendous strain on these patients, and this allows them to … get out of the four walls of the Critical Care.” (RT)
Early rehabilitation affects duration of mechanical ventilation. (25)	“For me, ventilator days. If we’re seeing a … significant decrease in ventilator days in our patient population I think that would … go a long way.” (RT)
Environmental context and resources domain	
We have adequate staff to perform early rehabilitation. (35)	“Staffing is our main thing; that is a huge thing which interferes with… what we want to do.” (PT)
ICU specialized equipment is required for early rehabilitation. (30)	“We really just need walkers. We don’t use anything special. I know people have fancy stuff; we don’t.” (PT)
Early rehabilitation requires coordination and scheduling between staff and team members. (22)	“The problem is that it’s a multidisciplinary process so it does involve … all the RTs, all the nurses, all the physios, the dieticians, so it involves everybody. To get everybody to organize to do anything is always a challenge.” (RT)
Early rehabilitation requires therapy staff specifically assigned to the ICU. (16)	“I would definitely say that even amongst the physio personnel it would be better to either maintain a smaller, more experienced group continually coming to the ICU.” (RT)
Social influences domain	
Local champions influence early rehabilitation practice. (12)	“Our lead physical therapist recently left and she was a huge advocate of mobilizing patients and it quickly became apparent how person-specific our mobility culture was, that she was driving a lot of it.” (MD)
ICU leadership facilitates early rehabilitation practice. (14)	“It wouldn’t happen without the leaders of the ICU, the lead nurses and the nurse educators.” (PT)
Discord between team members affects delivery of early rehabilitation. (30)	“…we go in there and they [say], don’t touch them, they are finally settled. Don’t touch them … they’re sleeping. Don’t touch them; they have a line in them. And I’m like, yeah so what? So, it can definitely influence things.” (PT)
Family members affect delivery of early rehabilitation. (33)	“I think for the most part we’re probably undershooting the goals, so we’re actually doing less in order to not … freak out the family.” (RT)
Behavioral regulation domain	
Feedback affects early rehabilitation practice. (33)	“I think talking about the successes and failures and how we could make it better would be more important. I don’t think we get as much feedback on that as I think would be beneficial to say, hey, this is working and this is where we fell short and we need to step up to do a better job.” (RT)
Having a unit protocol facilitates early rehabilitation.(27)	“It would be nice to have a standard of care with regard to at least a consideration of mobilization and maybe realize that everybody will need to make their own decisions, but we’re asking a question; has the patient mobilized and if not, what sort of barriers or what sort of thought processes getting in the way of that happening should be undertaken?” (PT)

*RN* nurse, *MD* physician, *RT* respiratory therapist, *PT* physiotherapist, *OT* occupational therapist

### Skills

Participants reported that early rehabilitation was facilitated by working with experienced colleagues.

### Social/professional role and identity

Underscoring the fact that early rehabilitation is a complex, team level behavior, a large number of specific roles were identified for multiple team members. In particular, physician roles as team leaders and as those who identify appropriate patients for rehabilitation were identified, although most frequently by the physician group rather than by other groups. The importance of a general “leadership role” for physicians was emphasized by all professional groups.

### Beliefs about capabilities

Most participants reported that early rehabilitation was a difficult therapy to deliver (a potential barrier); however, some felt that it was fairly “easy” and fell within their skill sets as ICU clinicians.

### Beliefs about consequences

Participants reported a broad range of benefits of early rehabilitation. In particular, improved strength or muscle mass, improved long-term function, improved mental health, and shorter duration of mechanical ventilation were identified as important.

### Environmental context and resources

There was a range of views about the adequacy of staffing for early rehabilitation; some reported under-staffing as a barrier, while some felt staffing was adequate. There was similar diversity of views about whether “specialized” equipment was a facilitator. However, there were similar views both across and within professional groups that coordinating the various staff members and equipment needed at a time that was optimal for a patient was a barrier. There was also consistency in the belief that a model for early rehabilitation with physiotherapists specifically assigned to the ICU, rather than a rotating model was a facilitator.

### Social influences

There was a frequently held view that local “champions” facilitated early rehabilitation. In addition to local champions, it was also reported by 6/10 physicians, 4/10 nurses, and 4/10 physical therapists, and none of the respiratory therapists that the support of ICU leadership was important. Family members were reported by all professional groups to influence early rehabilitation, although sometimes as a facilitator and sometimes as a barrier. All participants reported that discord or resistance from colleagues could be an important barrier to early rehabilitation.

### Behavioral regulation

The importance of receiving feedback about early rehabilitation as a facilitator was noted by all groups; however, there was a range of views about whether or not feedback was actually received (a potential barrier). A unit protocol to guide early rehabilitation practice was reported by all groups as a facilitator.

### Differences between professional groups

Quantitative differences in stated beliefs about early rehabilitation were not common (see Additional file 6). Eighteen of the 59 beliefs (31%) of at least moderate importance showed evidence of a difference in frequency between groups. In most cases (13/18, 72%), physicians were one of the two groups who differed. Most of the beliefs (13/18, 72%) fell under one of either social/professional role and identity, skills, or social influences. With social/professional roles, the differences were largely related to the roles of the participants assigned to their own professional group. For example, physicians frequently reported that they were responsible for goal setting, whereas physiotherapists did not identify this as a physician role.

The majority of physiotherapists reported the importance of practical experience for development of skills (7 of 10) compared with only 2 of 10 in each of the other professional groups (skills domain). Physicians (6 of 10) and nurses (5 of 10) reported the importance of “local champions” in early rehabilitation. In contrast, no members of the physiotherapy group reported this and only 1/10 of the respiratory therapy group did.

## Discussion

This study used the TDF to study the beliefs of ICU clinicians regarding the barriers and facilitators to early rehabilitation in mechanically ventilated patients. We identified seven domains of the TDF which were most relevant to the behavior of clinicians and found that differences between clinician groups were uncommon. While the domains of environmental context and resources, as well as beliefs about consequences, are commonly identified in existing barriers literature [33–37], this approach facilitated a broader view of barriers and facilitators than prior literature. In particular, we demonstrated important factors not previously emphasized in the literature, in particular in the domains of social influences and behavioral regulation, which are novel findings in this field. These domains should be used to specifically direct implementation and quality improvement efforts.

For example, a recent cross-sectional study of hospital factors that influence early rehabilitation demonstrated that a formal protocol for early rehabilitation was associated with increased uptake [38], a strategy which falls under the domain of behavioral regulation. In a non-randomized interventional study, Hanekom et al. demonstrated that the introduction of a protocol for early rehabilitation increased frequency of rehabilitation sessions and reduced waiting time [39]. These findings, combined with our study showing the importance of the behavioral regulation domain, suggest that using a formal protocol to support early physical rehabilitation in the ICU setting may be helpful.

The domain of social influences, which we identified as important, is less frequently identified in the literature as a facilitator, although sometimes identified as a negative influence. We would suggest studies exploring specifically the role of “local champions” as a starting point, since our participants identified this as a useful facilitator.

Using a theoretically driven strategy provides potential for linkage to interventions for behavior change. Michie et al. have identified behavior change techniques and mapped them to theoretical domains as a starting point for the development of interventions [40]. For example, leveraging local opinion leaders may be a useful intervention to target the social influences domain [41].

A second advantage is to identify those domains that are less important so that efforts and resources can be focused away from those areas. For example, the knowledge domain was not found to contain a high number of beliefs in this study. Although the study sample was composed of volunteers and therefore may not be representative of all clinicians, this finding is consistent with prior knowledge translation research, which has shown only modest effects of educational interventions on clinician behavior [42–44].

The importance and level of elaboration of the domain social/professional role and identity was noteworthy in this study. TDF studies that investigate clinical behavior most often report beliefs about consequences (reflecting clinical thinking in terms of the balance between benefits and risks) as the most populated domain [45]. The importance of professional role in the current study provides a clear indication that team work and role clarity may be key to the implementation of early rehabilitation.

This study has a number of limitations. First, participants were volunteers recruited from online interest groups and professional organizations, which may create selection bias. A high number of participants reported determination to engage in early rehabilitation (intentions domain). In addition, within the domain of beliefs about consequences, participants endorsed a high number of specific positive associations with early rehabilitation. While supportive evidence exists, some of the specific beliefs endorsed by participants are not supported in the literature (e.g., mortality benefit). In addition, there was a commonly held belief that future literature would demonstrate further benefit (optimism domain). Participants may have experienced the equivalent of a “halo effect” [46], where a generally positive view of early rehabilitation creates a cognitive bias leading to other positive beliefs about early rehabilitation which may not be supported by evidence.

A second limitation is in the method by which we identified important beliefs and important domains. Our interviews generated a large volume of data, and it was necessary to try to identify those domains that were most important, in particular with the view that targeted interventions should be focused on those barriers and facilitators most likely to impact on early rehabilitation. We used a variety of methods to identify important domains based on work in prior literature [32], but it is not yet established that this method will lead to more successful interventions.

## Conclusions

Using a theoretically driven approach, this study identified important barriers and facilitators to early rehabilitation in ICU patients. In particular, the domains of social influences and behavioral regulation were not previously well described in the literature. Future interventions should include interventions targeted at these domains, such as the institution of formal protocols to guide physical rehabilitation in the ICU. Differences between professional groups were uncommon but, where they exist, highlight the importance of involving an inter-professional team in implementation. Further work is required to validate our method for identifying importance and to determine the frequency of barriers and facilitators in other stakeholder groups.

## Additional files

Additional file 1: Domains of the Theoretical Domains Framework. (DOCX 13 kb)

Additional file 2: Topic Guide. (DOCX 16 kb)

Additional file 3: Generation of TDF Domains and Beliefs. (DOCX 19 kb)

Additional file 4: Criteria for Evaluating Importance of Beliefs. (DOCX 69 kb)

Additional file 5: Beliefs of Moderate Importance. (DOCX 30 kb)

Additional file 6: Frequency of all Beliefs by Profession. (DOCX 29 kb)

## Abbreviations

ICU: Intensive care unit
IRN: ICU Recovery Network
TDF: Theoretical Domains Framework
