# Supplementary material for: Barriers and facilitators to early rehabilitation in mechanically ventilated patients—a theory-driven interview study
Source: J Intensive Care. 2018 Jan 23;6:4. doi: 10.1186/s40560-018-0273-0 (PMC5781271; doi:10.1186/s40560-018-0273-0)
Supplement: Supplementary file 1 — Domains of the Theoretical Domains Framework. (DOCX 13 kb) [file 40560_2018_273_MOESM1_ESM.docx]

**Additional File 1. Domains of the Theoretical Domains Framework**

| **Domain** | **Description** | **Example Question From Topic Guide** |
| --- | --- | --- |
| Knowledge | Knowledge/awareness of the scientific rationale, evidence base and how to perform the behavior | Are you aware of any hospital or unit guidelines or clinical protocols regarding early rehabilitation for ICU patients in your institution? |
| Skills | Ability to perform the behavior of interest, including safety, use of equipment. May include non-technical skills. | What skills are needed for you to undertake early rehabilitation with ICU patients? |
| Social/profes-sional role | Belief about one’s own role, as well as the role of others in the target behavior. | To what extent does early rehabilitation fit with your professional role as a doctor/nurse/etc? |
| Beliefs about capabilities | The degree to which a clinician feels he/she/the team can perform the target behavior | How confident are you in undertaking early rehabilitation with an ICU patient? |
| Optimism | Belief that things will turn out for the best. | In general, how important do you think early rehabilitation will be in the care of ICU patients in the future? |
| Beliefs about consequences | The influence of expected positive or negative outcomes of the behavior. May include outcomes for others (e.g. patients) or for clinicians. | What do you see as the benefits of undertaking early rehabilitation? |
| Reinforcement | Strategies to change likelihood of behavior by making it contingent on reward or punishment. | Are you aware of any consequences for NOT undertaking early rehabilitation? |
| Intentions | The degree to which the individual means to perform the behavior. | How determined are you to engage in early rehabilitation with ICU patients? |
| Goals | The end states that the behavior is meant to achieve. | How are goals for early rehabilitation set? Are they explicitly stated? |
| Memory, attention and decision processes | The ability to pay attention to relevant information and make appropriate decisions. | How are decisions made in your unit about which ICU patients receive early rehabilitation? |
| Environmental context and resources | The influence of the environment on the individual’s performance of the behavior – includes culture, human and other resources | What physical setup of the ICU is best for early rehabilitation? How close is your ICU to the ideal setup? |
| Social influences | The influence of others (health care providers, experts, patients, etc) on the behavior. | To what extent do the views or practices of other team members influence how you undertake your role in early rehabilitation? |
| Emotion | The role of both positive and negative emotions on performance of the behavior. | To what extent does undertaking early rehabilitation affect you emotionally? |
| Behavioral regulation | Purposeful strategies to overcome obstacles to behavior. | Are there strategies to improve early rehabilitation when goals are not met? |
