# Supplementary material for: Barriers and facilitators to early rehabilitation in mechanically ventilated patients—a theory-driven interview study
Source: J Intensive Care. 2018 Jan 23;6:4. doi: 10.1186/s40560-018-0273-0 (PMC5781271; doi:10.1186/s40560-018-0273-0)
Supplement: Supplementary file 2 — Topic Guide. (DOCX 16 kb) [file 40560_2018_273_MOESM2_ESM.docx]

**Additional File 2. Topic Guide:**

**1. How would you describe “early rehabilitation” for ICU patients? *(Domain – Knowledge)***

- What does “rehabilitation” mean in this context? (synonyms, physiotherapy, mobilization, etc)
- What activities might qualify as early rehabilitation?
- What does “early” mean in this context? How long after ICU admission?

***For the purposes of this interview, we will use the term “early rehabilitation” to refer to physical rehabilitation activities that occur during the ICU stay, even while the patient is mechanically ventilated.***

***2.* In your view, what is the biological rationale for early rehabilitation? *(Domain – Knowledge)***

- How consistent is the evidence base with the biological rationale?

**3. What do you think is the evidence for early rehabilitation for ICU patients in the published literature? *(Domain – Knowledge)***

- Is there evidence for benefit?
- What about harm?
- How strong is that evidence?
- How credible is the evidence?

**4. Are you aware of any hospital or unit guidelines or clinical protocols regarding early rehabilitation for ICU patients in your institution? *(Domain – Knowledge)***

- Is there a document?
- Have you read the document?
- Are you aware of what they say?
- How credible do you think these guidelines/policies are?

**5. What specific roles do YOU play in undertaking early rehabilitation with ICU patients? *(Domain – Social/Professional Role and Identity)***

- To what extent does early rehabilitation fit with your professional role as a doctor/nurse/etc?

**6. What specific roles do other clinicians play in early rehabilitation? *(Domain – Social/Professional Role and Identity)***

- Which clinicians should be involved? In what parts of early rehabilitation should they be involved?
- What roles do the leaders within the unit play in facilitating early rehabilitation?

**7. How does undertaking early rehabilitation with ICU patients fit in with the other responsibilities of your job? *(Domain – Social/Professional Role and Identity)***

- Are there other activities that require your attention that interfere with early rehabilitation?
- How do you balance early rehabilitation with those other activities?

**8. How are decisions made in your unit about which ICU patients receive early rehabilitation? *(Domain – Memory, Attention and Decision Processes)***

- Who is involved?
- Is there a process or routine for this?

**9. How determined are you to engage in early rehabilitation with ICU patients? *(Domain – Intentions)***

- Are there things that interfere with your intentions?

**10. What skills are needed for YOU to undertake early rehabilitation with ICU patients? *(Domain – Skills)***

- To what extent do you feel you have these skills?
- How can these skills be developed?
- What facilitates the development of these skills?
- What interferes with the development of these skills?
- Are there other “non-technical” skills that are needed?
- Do other types of clinicians need the same skills for early rehabilitation?

**11. How confident are you in undertaking early rehabilitation with an ICU patient? *(Domain – Beliefs about Capabilities)***

- What things would improve your confidence?
- What things would improve your team’s confidence?
- What things would decrease your confidence?

**12. How easy or difficult do you find early rehabilitation with ICU patients? *(Domain – Beliefs about Capabilities)***

**13. What do you see as the most important goals in undertaking early rehabilitation with ICU patients? *(Domain – Goals)***

**14. How are goals for early rehabilitation set? *(Domain – Goals)***

- Who sets them?
- Are they explicitly stated?
- How are goals adapted in response to changes over time in the patient’s clinical status?
- How are goals adapted in response to events that occur during a physiotherapy session?

**15. What do you see as the benefits of undertaking early rehabilitation? *(Domain – Beliefs about Consequences)***

- Are there short-term benefits (e.g. during the ICU stay)?
- Are there long-term benefits (e.g. after the ICU stay or after the hospital stay?)
- Do these benefits differ according to different types of patients?
- What types of physical benefits are there?
- What types of cognitive (e.g. thinking, mental processing) benefits are there?
- What types of mental, psychological or psychiatric benefits are there?

**16. What do you see as the risks or adverse consequences that can occur as a result of undertaking early rehabilitation? *(Domain – Beliefs about Consequences)***

- Are there patient safety concerns?

**17. What resources are needed to undertake early rehabilitation? *(Domain – Environmental Context and Resources)***

- What equipment is needed?
- What physical setup of the ICU is best? (e.g. layout of the rooms, the unit)
- How close is your ICU to the ideal setup?
- To what extent do you feel your hospital already has the necessary levels of these resources?
- Are these resources consistently available and functioning when you need them?
- What staffing is required? Is your hospital staffing adequate?

**18. To what extent do the views or practices of other team members influence how you undertake your role in early rehabilitation? *(Domain – Social Influences)***

- Who else might influence you?

**19. To what extent do you feel the ICU culture encourages or discourages early rehabilitation? *(Domain – Environmental Context and Resources)***

**20. To what extent do patients and their family members influence whether you undertake early rehabilitation? *(Domain – Social Influences)***

**21. How do you prepare to undertake early rehabilitation? *(Domain – Behavioural Regulation)***

- Do you follow any action-plans? (If-then statements; where/when/who/how plans)

**22. How does your unit monitor your participation in early rehabilitation? *(Domain – Behavioural Regulation)***

- Who monitors? Are there specific “metrics” that you know of? Does anyone audit early rehabilitation?
- How often?

**23. What kind of feedback is given when early rehabilitation does not occur? *(Domain – Behavioural Regulation)***

- How is the feedback structured?
- Who gives the feedback?
- To whom is the feedback targeted? Individuals? Teams?
- What about when it occurs, but standards are not being met?
- Are teams or individuals held accountable when rehabilitation plans do not occur?
- How does this occur?

**24. Are you aware of any ways in which undertaking early rehabilitation is encouraged or rewarded in your unit? *(Domain – Reinforcement)***

- Is success celebrated or recognized?

**25. Are you aware of any consequences for NOT undertaking early rehabilitation? *(Domain – Reinforcement)***

- What sorts of consequences are there?

**26. Are there strategies to improve early rehabilitation when goals are not met? *(Domain – Behavioural Regulation)***

**27. To what extent does undertaking early rehabilitation affect you emotionally? *(Domain – Emotion)***

- Any feelings of stress, anxiety or depression?
- What about positive feelings?

**28. How does this affect how you undertake early rehabilitation? *(Domain – Emotion)***

- Why? Why not?

**29. In general, do you think your unit has the ability to improve the delivery of early rehabilitation? *(Domain – Optimism)***

- Why?

**30. In general, how important do you think early rehabilitation will be in the care of ICU patients in the future? *(Domain – Optimism)***
