# Supplementary material for: Barriers and facilitators to early rehabilitation in mechanically ventilated patients—a theory-driven interview study
Source: J Intensive Care. 2018 Jan 23;6:4. doi: 10.1186/s40560-018-0273-0 (PMC5781271; doi:10.1186/s40560-018-0273-0)
Supplement: Supplementary file 3 — Generation of TDF Domains and Beliefs. (DOCX 19 kb) [file 40560_2018_273_MOESM3_ESM.docx]

**Additional File 3. Generation of TDF Domains and Beliefs**

“I’ve found that when the physicians are really on board, everyone else will follow suit and get excited about mobility.”

Social Influences

TDF Domain

Belief

When physicians engage in early rehabilitation, they influence the rest of the healthcare team.

Hypothetical Excerpts

“Some doctors are very good about bringing up rehab goals on rounds but others ignore it. If they bring it up, it makes the nurses, physios and everyone else realize it’s just as important as all the medical details they’re managing.”
