# Supplementary material for: Barriers and facilitators to early rehabilitation in mechanically ventilated patients—a theory-driven interview study
Source: J Intensive Care. 2018 Jan 23;6:4. doi: 10.1186/s40560-018-0273-0 (PMC5781271; doi:10.1186/s40560-018-0273-0)
Supplement: Supplementary file 4 — Criteria for Evaluating Importance of Beliefs. (DOCX 69 kb) [file 40560_2018_273_MOESM4_ESM.docx]

Additional File 4. Criteria for Identifying Important Beliefs

| Description | Definition and Rationale |
| --- | --- |
| 1. Frequency of belief | Expressed by more than 50% of participants |
| 1. Participant expression of importance | Qualitative assessment by study team of phrases used to express importance (e.g. “it’s critical”, “the most important thing”). Specific statements meeting these criteria agreed on by in-person review and consensus. |
| 1. Discord among participants about belief as a barrier or facilitator | Qualitative classification by study team of a stated belief as both a barrier and facilitator . (e.g. a piece of equipment that was used facilitated early rehab or the lack of the equipment was a barrier) |
| 1. Inter-professional differences | Quantitative difference in frequency of an expressed belief between clinician groups of at least 5 participants |
| 1. Spontaneous vs. elicited belief | Spontaneously expressed beliefs (i.e. those not elicited by a topic guide question). |
