# Supplementary material for: Barriers and facilitators to early rehabilitation in mechanically ventilated patients—a theory-driven interview study
Source: J Intensive Care. 2018 Jan 23;6:4. doi: 10.1186/s40560-018-0273-0 (PMC5781271; doi:10.1186/s40560-018-0273-0)
Supplement: Supplementary file 5 — Beliefs of Moderate Importance. (DOCX 30 kb) [file 40560_2018_273_MOESM5_ESM.docx]

**Additional File 5. Beliefs of Moderate Importance**

| **Knowledge domain** | |
| --- | --- |
| Knowledge of protocols or guidelines impacts performance of early rehabilitation (22) | “So I know we have a protocol... and I follow it to some degree but honestly I don’t have it memorized.” (RN) |
| Education and knowledge facilitate early rehabilitation (28) | “I think once education is out, then we understand what type of rehabilitation needs to be done and why it’s important, then we can start to build on that and move forward, … it’s the first priority.” (RT) |
| Gaps exist in literature base. (15) | “There’s a lot of theory behind it and it makes probably intuitive sense but I still think it needs to have the definitive study put into place.” (MD) |
| **Skills Domain** | |
| Specialized skills are (or are not) required for early rehabilitation. (17) | “I feel like as ICU nurses we already have the knowledge and expertise, just what we do every day to also engage in early rehabilitation with our patients.” (RN) |
| Practical training is required to develop skills for early rehabilitation (13) | “… you bring them right into the ICU, with a mentor, and you just educate them and make them review all the lines and the tubes and everything else and what the numbers mean, and then actually just have them start to treat the patient with someone with good experience there.” (PT) |
| On the job experience develops skills needed for early rehabilitation. (14) | “I think the second is learning within your institution. … basically mobilizing the patients over time and learning skills and the way things work are important.” (MD) |
| Interpersonal skills are required for early rehabilitation (11) | “You’ve got to have people skills. You’ve got to put your patients at ease. They’re probably at one of their lowest, scariest points in their life and they’re trusting you that you’re going to come in and get them up” (PT) |
| Communication skills are needed for early rehabilitation (11) | “Good communication, making sure that we’re, … communicating with the team effectively, making sure everybody on the team’s on board, and being able to communicate the plan effectively.” (RT) |
| **Social / Professional Role and Identity** | |
| The physician’s role is not in the actual delivery of early rehabilitation. (6) | “I think that my role personally in the actual act of early rehabilitation is somewhat limited because … I’m not specifically trained in the physical activity of actually assisting patients in this, and I think that’s more the role of the physiotherapist, or potentially nursing” (MD) |
| The physician has a role in goal setting. | “…the main keeper – of the goals does end up being the primary physician for the week.” (RN) |
| The physician’s role is to advocate for and encourage early rehabilitation (9) | “From a clinical standpoint my role is to ask our physical therapist to evaluate and treat patients and then to kind of cheerlead our nurses to help facilitate mobility in ways that they can.” (MD) |
| The physiotherapist has a role in goal setting. (21) | “As a PT I’m making my own individualized goals for that patient. I have the care plan in mind but I’m thinking about what I think is realistic for that person.” (PT) |
| My other duties can conflict with early rehabilitation (11) | “…there are other things that I have to do. Documentation’s probably the number one thing. … hygiene, giving meds, taking vital signs, talking with family. … there’s lots of stuff I have to do that gets in the way …”  (RN) |
| Early rehabilitation is a team responsibility (21) | “So it’s really about teamwork, working independently but also working in that big team, and working what’s best with the nurse and working what’s best with the patient when they’re clinically able to move, when it’s safest for them, and when it’s best for the team to get that patient up.” (RT) |
| Nursing staff have an important or central role in early rehabilitation. (7) | “…in terms of the ICU team you know the bedside nurse has a huge role to play because essentially I think they’re the managers of the patient’s activities for the shift and really facilitate and drive the plan in terms of the mobilization.” (MD) |
| Charge or lead nurses have an important role to play in early rehabilitation. (8) | “I have worked in one [ICU] where the charge nurses did really promote it and go to each patient so having an actual charge nurse role and having a charge nurse have time to go out and assess each nurse and support them was always helpful.” (RN) |
| The respiratory therapist has an important role with patients on mechanical ventilation. (17) | “Respiratory therapy is a very huge, integral part of mobilization. They will often come in and help us to walk a patient who’s on a ventilator. The hoses need to be switched over. .… most of the time, they’ll require a larger amount of oxygen, a different rate. So the respiratory therapist will be on board with that.” (PT) |
| **Beliefs about Capabilities** | |
| The support of a team improves confidence in early rehabilitation (13) | “I got oriented by another PT but ultimately it was the nurses that helped me develop the bravery, I guess, in the beginning, and the attitude that … it could be done.” (PT) |
| Experience improves confidence with early rehabilitation.(17) | “When we first instituted it and were training people it was obviously a problem, but now that we have a long track record and experience and receive some extra staffing on the intensive care unit we’re very, very confident.” (MD) |
| Lack of confidence interferes with early rehabilitation. (28) | “I would say [I am] not confident. For those reasons, based on lack of understanding of how to do it safely, both on my part and on the part of people that I work with.” (MD) |
| **Optimism** | |
| I am optimistic research will show more benefits for early rehabilitation in the future. (6) | “I think the evidence is there and I think the strength of the evidence will probably get bigger with bigger studies, and I think in pushing the bar to mobilizing sicker patients we may start to see a greater impact on that population from early mobilization.” (MD) |
| Early rehabilitation will be part of ICU practice in the future. (23) | “I think it’ll be... well, they say it’s going to be A, B, C, D, E, exercise. You know, we’re all about airway, breathing, circulation, disability, and now E, I think it’s going to be just right up there.” (RT) |
| **Beliefs about Consequences** | |
| Benefits outweigh the harm for patients in early rehabilitation (32) | I’m not aware of any studies that support that getting a patient mobile early is going to do more harm than good.” (PT) |
| Early rehabilitation affects delirium (20) | “Delirium does not set in if you start to see them 24 hours to 48 hours after. And that’s very well documented in the literature, now.” (PT) |
| Early rehabilitation affects mortality (9) | “Everything that I've seen has suggested it's better from a mortality standpoint” (RT) |
| Early rehabilitation affects ICU length of stay (28) | “So I think there’s a growing body of evidence that supports that it’s helpful in shortening the ICU and hospital length of stay.” (MD) |
| Early rehabilitation can cause physiological deterioration (21) | “I think another major concern is if the patient, from a physiologic perspective, does not have the stamina or strength to engage in early mobilization and they’re pushed to do that that could lead to physiologic consequences such as a drop in their blood pressure or hypoxia” (MD) |
| Early rehabilitation is safe (32) | “Yes, well I think that goes along with if they lose any tubes and lines they have a high risk of complications as well. I mean they would be at a higher risk for falling, just getting out of the bed. But I think with the appropriate assessment and appropriate equipment and people, it is a safe thing to do.” (RN) |
| Early rehabilitation is personally rewarding to healthcare providers. (16) | “I find it satisfying … When you feel like you've done a good job with a patient, you feel proud and positive about what you're doing.” (RN) |
| **Reinforcement** | |
| There are [no] consequences in my unit for not participating in early rehabilitation. (29) | “Not to my knowledge. I wish there were! Sometimes I’m like, “What did this nurse do all day?” I’m sure they were busy, you know, but there are some nurses who I think they just... they’re just not entirely on board.” (PT) |
| **Intentions** | |
| I/we are determined to engage in early rehabilitation. (28) | “Oh, I'm very determined. I'm very determined. We’ve been doing this, like I said, for a year. I’ve been pushing and pushing and pushing.” (RN) |
| **Goals** | |
| The goal is to improve early rehabilitation within our unit. (14) | “I see our goal as putting together a working group to devise the process for early mobilization. I think that’s our short-term goal, is to actually put a process in place.” (RT) |
| Goals should be explicitly stated and reviewed. (23) | “The goals are not recorded to my knowledge. They might be stated on rounds but inconsistently.” (MD) |
| **Memory, Attention and Decision** | |
| Decisions about early rehabilitation are made as a team. (20) | “…it’s all a discussion between the whole team, the physicians, the physiotherapists and the nurse sort of as to deciding on what the plan is.” (RN) |
| **Environmental Context and Resources** | |
| ICU culture affects early rehabilitation (34) | “I’m really lucky because our culture is not just encouraging, it demands it, and I think... I talk to other people and it seems like it can be quite a battle if the culture is one of … bed rest.” (PT) |
| We have an adequate physical layout to perform early rehabilitation (34) | “Horrible. We have tiny rooms that are very bed-centric, and our ICU is shaped as a triangle, which is nice for visibility but when interdisciplinary rounds are occurring ….number of bodies on the unit is challenging, and sometimes it’s the role of the nursing assistant to encourage people to step aside during rounds.” (RN) |
| We have adequate equipment for performing early rehabilitation (28) | “Our beds are now 14 years old and often don’t function the way they’re supposed to and they don’t go down low enough.” (PT) |
| **Social Influences** | |
| Comparison with other team members practice impacts my rehabilitation practice. (16) | “Like, maybe there’s a level of sickness that I wouldn’t mobilize someone that a colleague would, and I think just understanding and seeing examples of that being done safely would help improve my confidence in achieving that.” (MD) |
| Physicians influence early rehabilitation practice. (15) | “If you have even one or two physicians that are against that it can surprisingly go awry.” (RN) |
| **Behavioral Regulation** | |
| We discuss early rehabilitation plans every day on rounds. (21) | “So, after you talk about all of the systems, talking about mobilization I think is important for every patient, and in some units they actually have a physiotherapist that rounds with the team. However, that’s not the case with all of the units and I think perhaps if there was the presence of the physiotherapist that may, from an optics standpoint, help remind the team about addressing the question of mobilization of that patient.” (MD) |
