# Supplementary material for: Barriers and facilitators to early rehabilitation in mechanically ventilated patients—a theory-driven interview study
Source: J Intensive Care. 2018 Jan 23;6:4. doi: 10.1186/s40560-018-0273-0 (PMC5781271; doi:10.1186/s40560-018-0273-0)
Supplement: Supplementary file 6 — Frequency of all Beliefs by Profession. (DOCX 29 kb) [file 40560_2018_273_MOESM6_ESM.docx]

Additional File 6- Frequency of all Identified Beliefs, by Professional Group

| **Domain/Belief** | **MD (N=10)** | **Rehab (N=10)** | **Nurse (N=10)** | **RT (N=10)** |
| --- | --- | --- | --- | --- |
| **Knowledge** | | | | |
| Knowledge of protocols or guidelines impacts behaviour | **6** | **4** | **7** | **5** |
| Education and knowledge facilitates early rehabilitation | **10** | **5** | **8** | **5** |
| Gaps exist in literature base of early rehabilitation | **6** | **5** | **3** | **1** |
| **Skills** | | | | |
| I have the skills needed for early rehabilitation | **2** | **9** | **5** | **2** |
| My team has the skills for early rehabilitation | **4** | **4** | **4** | **2** |
| Skills or no specific skills are needed for early rehabilitation | **7** | **3** | **3** | **4** |
| Biomechanic skills are required for early rehabilitation | **0** | **1** | **3** | **2** |
| Managing awake patients in the ICU is a required skill for early rehabilitation | **1** | **0** | **0** | **1** |
| Knowledge of how to manage equipment is required for early rehabilitation | **1** | **1** | **0** | **0** |
| Physical strength is required for early rehabilitation | **0** | **2** | **1** | **0** |
| Practical training is required to develop skills for early rehabilitation | **2** | **7** | **2** | **2** |
| On the job experience develops skills needed for early rehabilitation | **3** | **6** | **4** | **1** |
| Development of skills for early rehabilitation requires institutional support | **0** | **1** | **1** | **0** |
| Time limits interfere with skills development for early rehabilitation | **2** | **2** | **1** | **1** |
| Skills for early rehabilitation are developed by working with experienced colleagues | **5** | **5** | **2** | **0** |
| Time management skills are needed for early rehabilitation | **0** | **1** | **0** | **0** |
| Communication skills are needed for early rehabilitation | **2** | **7** | **1** | **1** |
| Interpersonal skills are needed for early rehabilitation | **2** | **5** | **3** | **1** |
| Leadership skills are needed for early rehabilitation | **3** | **0** | **1** | **0** |
| Data analysis skills | **1** | **0** | **0** | **0** |
| **Social and Professional Role/Identity** | | | | |
| The charge nurses have leadership roles in early rehabilitation | **1** | **1** | **5** | **1** |
| Early rehabilitation is part of my role | **5** | **4** | **2** | **1** |
| Early rehabilitation is everyone's job | **3** | **1** | **1** | **0** |
| Early rehabilitation is a team responsibility | **4** | **7** | **6** | **4** |
| My other duties can conflict with early rehabilitation | **4** | **2** | **1** | **4** |
| My role is to set goals | **1** | **1** | **1** | **1** |
| Leadership role in implementing rehabilitation | **9** | **5** | **5** | **3** |
| MD role identifying and screening patients | **8** | **4** | **1** | **3** |
| MD role is as leader | **5** | **1** | **1** | **0** |
| MD role not in actual act of early rehab | **5** | **0** | **1** | **0** |
| MD role setting goals | **5** | **0** | **4** | **2** |
| MD role to educate | **4** | **0** | **0** | **0** |
| MD role to facilitate encourage advocate | **6** | **1** | **1** | **1** |
| MD role to write order | **5** | **3** | **4** | **1** |
| Mobility not a nurse's role | **0** | **0** | **1** | **0** |
| Mobilization is primarily a PT role | **1** | **2** | **1** | **1** |
| Nurse can be primary provider of rehab | **3** | **1** | **1** | **3** |
| Nurse has important or central role | **3** | **2** | **2** | **0** |
| Nurse has primary role in less sick or less complex patients | **2** | **3** | **0** | **0** |
| Nurse role coordinating around other patient care activities | **3** | **2** | **1** | **0** |
| Nurse role in carrying out the actual early rehab | **5** | **5** | **3** | **1** |
| Nurse role in monitoring patient | **1** | **2** | **1** | **0** |
| Nurse role setting goals | **5** | **1** | **3** | **1** |
| Nurse role to advocate | **1** | **0** | **0** | **1** |
| Nurse role to educate families | **1** | **0** | **1** | **0** |
| Nurse role to identify suitable patients | **4** | **3** | **4** | **2** |
| OT more involved with awake patients | **0** | **1** | **0** | **0** |
| OT role in ADLs | **1** | **2** | **0** | **0** |
| OT role in carrying out physical rehab | **0** | **2** | **0** | **0** |
| OT role in providing aids | **1** | **0** | **0** | **0** |
| OT role setting goals | **1** | **1** | **1** | **0** |
| OT role to identify appropriate patients | **1** | **0** | **0** | **0** |
| PT makes judgement about stopping physio session | **1** | **0** | **0** | **0** |
| PT role continues on ward after patients leave ICU | **1** | **0** | **0** | **0** |
| PT role in sicker or more complex patients | **1** | **2** | **0** | **1** |
| PT role setting goals | **7** | **6** | **4** | **4** |
| PT role to assess safety | **2** | **0** | **1** | **1** |
| PT role to carry out rehab | **5** | **5** | **3** | **2** |
| PT role to identify appropriate patients | **4** | **5** | **2** | **0** |
| PT rounds with team | **4** | **1** | **0** | **0** |
| RT role in facilitating rehab in ventilated patients | **7** | **3** | **3** | **4** |
| RT role setting goals | **0** | **0** | **0** | **1** |
| RT role to identify appropriate candidates | **2** | **0** | **1** | **1** |
| Unit assistants play role | **1** | **0** | **1** | **0** |
| **Beliefs about Capabilities** | | | | |
| Early rehabilitation is or is not challenging | **2** | **8** | **6** | **6** |
| Experience facilitates confidence in early rehabilitation | **3** | **6** | **5** | **3** |
| We can improve the delivery of early rehabilitation | **5** | **5** | **4** | **5** |
| General confidence in early rehabilitation | **7** | **8** | **5** | **8** |
| Team support facilitates confidence in early rehabilitation | **3** | **3** | **4** | **3** |
| Patient adverse events decrease confidence in early rehabilitation | **3** | **5** | **3** | **5** |
| Protocols improve confidence in ER | **1** | **0** | **0** | **0** |
| **Optimism** | | | | |
| Early rehabilitation will be in ICU practice in the future | **8** | **3** | **7** | **5** |
| Future research will show more evidence of the benefits of early rehabilitation | **4** | **1** | **1** | **0** |
| **Beliefs about Consequences** | | | | |
| Benefits outweigh the harm for patients in early rehabilitation | **10** | **7** | **7** | **8** |
| Early rehabilitation affects muscle mass or strength (or decreases atrophy or weakness) | **10** | **6** | **8** | **7** |
| Early rehabilitation affects long term physical function | **7** | **7** | **4** | **4** |
| Early rehabilitation affects long term cognition | **5** | **2** | **2** | **3** |
| Early rehabilitation affects use of sedating agents | **2** | **2** | **3** | **1** |
| Early rehabilitation affects patient wakefulness | **3** | **1** | **2** | **1** |
| Early rehabilitation affects the mental health of the patient | **8** | **6** | **7** | **6** |
| Early rehabilitation affects delirium | **4** | **6** | **7** | **3** |
| Early rehabilitation affects mortality | **5** | **2** | **0** | **2** |
| Early rehabilitation affects ICU length of stay | **7** | **8** | **6** | **7** |
| Early rehabilitation affects duration of mechanical ventilation | **9** | **4** | **5** | **7** |
| Early rehabilitation affects nosocomial complications | **4** | **5** | **4** | **3** |
| Early rehabilitation affects cost | **2** | **2** | **2** | **1** |
| Early rehabilitation causes distress to patients | **1** | **0** | **1** | **1** |
| Early rehabilitation can cause physiological deterioration | **6** | **6** | **5** | **4** |
| Early rehabilitation is safe | **10** | **8** | **7** | **7** |
| The benefits of early rehabilitation are different across patients | **4** | **4** | **5** | **2** |
| Early rehabilitation affects unit culture | **1** | **0** | **0** | **0** |
| Early rehabilitation changes family perceptions of ICU care | **4** | **2** | **1** | **1** |
| Seeing patient progress as a result of early rehabilitation is personally rewarding | **2** | **5** | **4** | **5** |
| Can cause injury to staff | **0** | **1** | **0** | **1** |
| Gives patient sense of accomplishment | **2** | **3** | **0** | **2** |
| Prevents readmission to ICU | **0** | **1** | **0** | **0** |
| **Reinforcement** | | | | |
| There are consequences for not participating in early rehabilitation | **6** | **6** | **8** | **9** |
| Participation in early rehabilitation is encouraged by colleagues | **2** | **3** | **6** | **3** |
| **Intentions** | | | | |
| I or we are determined to engage in early rehabilitation | **8** | **5** | **6** | **9** |
| **Goals** | | | | |
| Goal is unique to each patient | **4** | **5** | **2** | **1** |
| Goal to improve early rehab in our unit | **7** | **1** | **4** | **2** |
| Goals in the ICU short term not long term | **0** | **1** | **0** | **0** |
| Goals should be stated and reviewed | **7** | **6** | **6** | **4** |
| We have a process for setting goals | **4** | **3** | **6** | **3** |
| **Memory, Attention and Decision** | | | | |
| You need to pay attention to identify early rehabilitation candidates | **1** | **1** | **2** | **2** |
| Decision about early rehabilitation for individual patients are made by the team | **6** | **4** | **3** | **7** |
| We have to prioritize early rehabilitation against all other tasks | **3** | **3** | **2** | **4** |
| We have no standard process for decisions about early rehabilitation for individual patients | **1** | **3** | **0** | **1** |
| **Environmental Context and Resources** | | | | |
| ICU culture affects early rehabilitation | **9** | **9** | **7** | **9** |
| Sedation practices affect early rehabilitation | **2** | **1** | **3** | **2** |
| We have adequate staff to perform early rehabilitation | **10** | **10** | **6** | **9** |
| We have an adequate physical layout to perform early rehabilitation | **10** | **9** | **6** | **9** |
| ICU specialized equipment is required for early rehabilitation | **8** | **9** | **6** | **7** |
| We have adequate equipment for performing early rehabilitation | **6** | **10** | **5** | **7** |
| Early rehabilitation requires coordination and scheduling between staff and team members | **4** | **6** | **4** | **8** |
| Funding for early rehab is adequate. | **4** | **0** | **2** | **1** |
| Early rehabilitation requires dedicated ICU rehab staff | **4** | **5** | **4** | **3** |
| **Social Influences** | | | | |
| Hearing what works well at other institutions affects my early rehabilitation practices | **4** | **6** | **3** | **4** |
| My early rehabilitation practice is influenced by exposure to experts in the field | **2** | **2** | **0** | **1** |
| Guidelines from my professional organization impact early rehabilitation | **0** | **1** | **0** | **0** |
| Local champions influence early rehabilitation practice | **6** | **0** | **5** | **1** |
| ICU leadership facilitates early rehabilitation practice | **6** | **4** | **4** | **0** |
| Patients influence early rehabilitation | **4** | **6** | **4** | **5** |
| Family members influence early rehabilitation | **8** | **9** | **8** | **8** |
| Discord between professions affect early rehabilitation for individual patients | **6** | **9** | **7** | **8** |
| Comparison with other team members practice | **5** | **4** | **6** | **1** |
| Non-ICU physicians influence early rehabilitation practices in the unit | **0** | **3** | **0** | **0** |
| Physicians have a special role as advocates for early rehabilitation | **3** | **4** | **4** | **4** |
| **Emotion** | | | | |
| Fatigue affects participation in early rehabilitation | **0** | **2** | **0** | **0** |
| Fear affects participation in early rehabilitation | **4** | **3** | **3** | **0** |
| **Behavioural Regulation** | | | | |
| Formulating a personal action plan facilitates my early rehabilitation practice | **1** | **9** | **2** | **3** |
| We discuss early rehabilitation plans everyday on rounds | **7** | **4** | **7** | **3** |
| We receive feedback on early rehabilitation in my unit | **9** | **10** | **6** | **8** |
| Meetings improve the delivery of early rehabilitation | **6** | **6** | **3** | **4** |
| A unit protocol facilitates early rehabilitation practice | **8** | **6** | **5** | **8** |
